# Understanding the prescription of antidepressants: a Qualitative study among French GPs

**DOI:** 10.1186/1471-2296-12-99

**Published:** 2011-09-24

**Authors:** Alain Mercier, Isabelle Auger-Aubin, Jean-Pierre Lebeau, Paul Van Royen, Lieve Peremans

**Affiliations:** 1 Department of General Practice, Rouen University and CIC Inserm 0204, Rouen University, Rouen, France; 2 Department of General Practice, Denis Diderot Paris 7 University, Paris, France; 3 Department of General Practice, Tours University, Tours, France; 4 Department of Primary and Interdisciplinary Care, Faculty of Medicine, University of Antwerp, Antwerp, Belgium; 5 Department of Public Health, Vrije Universiteit of Brussels, Brussels, Belgium

## Abstract

**Background:**

One-tenth of France's population is prescribed at least one antidepressant, primarily by General Practitioners. The reasons for this high prescription rate remain unclear. One-third of these prescriptions may not comply with clinical practice guidelines, and 20% are potentially unrelated to any psychiatric condition. Our aim was to explore how GPs declare they use antidepressants in daily practice and understand their reasons for prescribing them.

**Method:**

Six focus groups including a total of 56 rural and urban GPs, with four interviews were performed. The topic guide focused on reasons for prescribing antidepressants in various primary care situations. Phenomenological analysis was performed by four researchers.

**Results:**

Antidepressants were seen as useful and not harmful. Personal assessment based on experience and feeling determined the GPs' decisions rather than the use of scales. Twenty-four "non-psychiatric" conditions possibly leading to prescription of antidepressants in primary care were found.

**Conclusions:**

The GPs reported prescribing antidepressants for a wide range of conditions other than depression. The GPs' decision making process is difficult and complex. They seemed to prefer to focus on their difficulties in diagnosing depression rather than on useless overtreatment. Instead of using the guidelines criteria to detect potential cases of useful prescription, physicians tend to use their own tools based on gut feelings, knowledge of the patient and contextual issues.

## Background

According to the French health insurance system, approximately 10% of the country's population is reimbursed for at least one antidepressant (AD) [1,2]. Between 1980 and 2008, AD sales increased sevenfold, from € 84 million to € 525 million [3]. Reports from the national insurance system and the European Commission have confirmed these trends [4,5]. France's consumption of ADs ranks amongst the highest in the European Union [5]. They are mainly prescribed by General Practitioners (GPs) [6]. The reasons for this high prescription rate by French GPs remain unclear. According to the available data, this consumption is supposed to be related to a higher rate of depressed patients in France, an increase in the number of patients treated for depression, and higher demand for medical care [6,7]. Clinical practice guidelines in France officially recommend using an AD for major depressive episodes and for anxiety only when the condition has consequences on the patient's life [8]. Dysthymia, isolated symptoms not fulfilling the DSM IV criteria, light or moderate episodes, or those lasting less than 15 days should not be treated by ADs. One-third of the prescriptions (30-35%) probably do not comply with these statements and 20% may not be related to any psychiatric evidence based on standardized questionnaires [9,10]. Furthermore, the data are not homogeneous: other studies have shown that in 85% of cases, the patients treated with GP-prescribed antidepressants met DSM-IV depression criteria [11]. The main indications for ADs are major depressive episodes and anxiety disorders. Other conditions, such as neuropathic pain, also result in prescription [12], but some indications remain controversial [13,14]. Similar trends have been noticed in other countries [15,16]. The aim of this study was to explore GPs' viewpoints regarding their reasons for prescribing antidepressants and to determine what indications they reported using them for in daily practice.

## Methods

### Participant recruitment procedure

Groups were chosen using a purposeful sampling method. This study population was drawn from two different sources. Firstly, four focus groups (FG) were held with GPs recruited from existing "quality circles". These GPs already knew each other from previous professional training sessions based on the French "peer group" technique. In this technique one of the participants presents to the group of GPs a structured analysis of patient files with a specific medical condition (e.g. diabetes, medications for high blood pressure). The group analyses all of the issues, decisions or potential improvements to the care of the patient and provides feedback to the participant presenting the case. The dynamic interaction amongst these group members enabled us to collect reliable data on their actual practices. Secondly, we organized two focus groups with locum GPs. Locum physicians could have a different, more critical point of view on the diagnostic and therapeutic options of the doctors they stand in for: They were taught about the importance of the guidelines during their recent initial medical training. All of the locums were replacing either rural or urban GPs and not only private-practice GPs (GP principals) involved in the peer groups. The option of running FG with locums was chosen in order to disclose attitudes or facts that the initial prescriber may have concealed. The first group of locums were invited using purposeful sampling and the second by a local association of locums. Four of the locum participants were unable to take part in the focus group and were invited for a semi-structured interview using the same topic guide. All participants were sent an invitation by the group leader, and also received a telephone call informing them about the general subject of the focus group session. The participants gave their informed consent before participating. According to the rules of the research centre, ethical approval was not necessary for this study. Five focus groups were held in Normandy (two in rural areas), and the other took place in a northern suburb of Paris. Overall, six focus groups were held, using a continuous process of collection and analysis. We ensured that the focus groups included younger and older, more experienced male and female private-practice GPs and locums from both urban and rural areas. Data saturation was reached after 4 focus groups with GP principals, and the second focus group with the locums did not bring any additional material. Information on participant profiles is detailed in the "results" section.

### Data collection

Qualitative data were collected through focus groups and interviews with GPs. The focus groups were conducted between November 2008 and April 2009. A topic guide was created based on the existing literature [17] and the researchers' previous experiences [18]. The interviews focused on the GPs' experiences, circumstances, influences, justifications, and explanations, concerning both the psychiatric and non-psychiatric conditions for which the antidepressants were described. Two trained moderators briefly explained the aim of the study and led the focus groups, using a detailed topic guide (table 1). The sessions began by assessing the difficulty of taking care of certain patients based on guidelines. The relevance of the GPs' prescriptions was never challenged. The participants were asked to share their personal experiences in dealing with antidepressant drugs. The moderator ensured that all issues were covered and that everyone participated. One researcher observed the group members, without intervening, and gathered information on nonverbal communication and the interaction between the participants. Audio recordings of all of the focus groups were made, and these recordings were later transcribed.

**Table 1:** Topic guide

Central focus	To discover and understand actual prescription of antidepressants in general practice
**Aim**	To explore the reasons for prescribing antidepressants in 2 main groups: in different settings (primary care situations) and in different diagnoses (primary care conditions)

**Instructions for the moderator**	

**Beginning the focus group**	-Ice breaking question: "Primary care context and GPs' expertise allow them to sometimes justifiably prescribe drugs in situations other than those assessed using official criteria. Could you tell us more about how you use ADs in primary care? -Do not let the GPs repeat the guidelines or take the "official" stance -Don't be afraid to let them discuss strange or extreme situations

**Questions about deciding on AD prescription in primary care situations**	-In your real practice, regarding prescription of ADs, how helpful are the major depression criteria? (e.g. DSM IV)? How do you use official scales to assess patients? How do you manage to decide in real life situations? -Could you explain in what way the context (e.g. social, family, work) of the patient influences your prescription? -What is your opinion about the efficacy of ADs? Do you think GPs "anaesthetize" their patients with ADs? -Do you feel justified in prescribing ADs in situations that do not fit in with the official rules (e.g. guidelines)? Could you explain why? How do you decide?

**Questions about primary care conditions leading to AD prescription**	-Do you ever prescribe antidepressants when the patient is not depressed or anxious? Could you explain your reasons for this? -For what kind of conditions would you prescribe this way?

**Examples for taking the discussion further**	-You often talk about "feeling". Could you tell us more what you mean by this? -Some of you talked about "real depression". Could you clarify this concept? -Locum physicians: What's your opinion on the other GPs' prescription of ADs?

### Analysis

A phenomenological approach was used. The first aim of this approach was to gather material coming from GPs personal experiences in their own real situations. Secondly, the analysis focused on what doctors said about themselves or their peers regarding their experiences with prescribing ADs. We gathered information on what the GPs said regarding the social, family and professional situations of their patients and on how they saw the prescription of ADs helping them to solve their patients' problems. The final aim was to identify the background and disease conditions that influenced the doctors' decision to prescribe. QSR Nvivo 8.0 Software was used to perform the analysis.

In practice, information on non-psychiatric diagnoses and situations other than those based on guideline criteria that lead to prescription of an antidepressant was brought together. Firstly, the researchers listened to the audio recordings and noted any emerging themes. Secondly, working on the written transcription, each researcher created a code list without any pre-conceived framework (open coding). These codes were shared and discussed within the research team. Units of information (codes on situations, e.g. diseases and social conditions) and concepts (e.g. the doctor's role, the doctor as a medication and placebos) were labelled. This information was examined and cross-analyzed with semantic indicators of doubt, insistence and certainty. The codebook was continuously revised, with the researchers comparing all of the codes in the event of disagreement and attempting to clarify their meaning, going back to the context until mutual consent was reached. Following an axial coding process, a matrix was developed in order to highlight the GPs' opinions about ADs in these various situations and to focus on the decision-making process.

## Results

### Participant profiles

The characteristics of the participants and practices are summarized in table 2.

**Table 2:** Characteristics of the 56 GPs interviewed for data collection

Physician characteristics		
	**Range**	**Mean**

**Age of GPs (n = 56)**	25 to 65	40

**Male/Female**	39/17	69/31

**Age of private practice GPs (n: 34)**	27 to 65	53

**Age of locum GPs (n = 22)**	25 to 32	27.5

**Number of years of practice (private practice GPs) (n = 34)**	4 to 35	20

**Number of years of practice locum GPs (n = 22)**	<1 to 8	3.5

**Practice characteristics**		

	**Number (n)**	**Rate (%)**

**Urban practice**	15	26

**Rural practice**	12	22

**Urban & rural practice**	28	50

**Teacher or tutor**	16	28

**Particular interest **(acupuncture, sports medicine)	2	3.5

### Key points

First of all, the expected usefulness of AD prescription for several medical conditions is presented. Many conditions result in prescription, which is seen as effective but generally only with a symptomatic effect. Secondly, the GPs' options for assessing patients and situations are presented. We will see how GPs cope with patient demands, struggling through difficulties and inventing a personal approach.

#### Usefulness of AD prescription (table 3)

**Table 3:** Usefulness of ADs

*Quote 1*	Self confidence	*"No difficulties to prescribe, I don't' find it difficult.. don't ask myself any existential questions"(FG 4 Male GP, 50, mixed practice)*
*Quote 2*	Safe and secure	*"Antidepressants are useful, and change our patients' lives completely ... They are safe drugs, with plenty of indications, very little dependence, ...people can still work and drive"..."prescribing them only in characterized major episodes, would be tragic for patients..." (FG 3, Male GP, 65, urban practice) " Now we have the SSRI and there is no problem handling the treatment"(FG 3 Male GP, 65, urban practice)*

*Quote 3*	Useless	*"What is the real effect of the AD? Watchful waiting probably accounts for 50% of the success of the treatment, (FG 5, Male GP, 27, locum)*

*Quote 4*	Risk	*"I haven't seen a suicide in 10 years. Before, it was terrifying" (FG 3, Male GP, 62, urban practice)*

*Quote 5*	Major Shift	*"When you lived with those old drugs, this new period is absolutely fantastic"(FG 1 Male GP, 60, rural practice)*

*Quote 6*	General use	*" You can use them amongst young and old people, workers, unemployed, housewives, it would be a mistake not to treat all of these moderate episodes, (FG 3 Male GP, 45, urban practice )*

*Quote 7*	Emergencies	*Feeling it is an emergency situation will not have any influence on my AD prescription. It will have an impact on my decision to call for an ambulance (FG2,Male GP, 50, rural practice)*

*Quote 8*	Indications	*"...Seen non-conventional prescriptions? It's never happened to me, I think it's exactly the contrary; many more patients should have an AD and they don't have one" (Interview 1, Female, 28, locum GP)*

*Quote 9*	Reality of (AD effect, of depression)	*He was so bad, and really improved a lot in 7 to 14 days, and of course you do know that is not only the "real" effect of ADs (FG 6, Female, 26, locum GP)*

*Quote 10*	GPs' posture	*"Moral suffering is by no means trivial! Psychiatrists giving us advice about GPs over-prescribing ADs. You have to laugh or else you cry"(FG 4, Male, 51, Mixed practice GP)*

*Quote 11*	Defense	*"What they say is we prescribe ADs to get rid of our patients, not listen to them, and it's exactly the contrary in our real daily practice" (FG 2 female GP, 48, Rural practice)*

*Quote 12*	Real effect	*"You always come back to the same question: are antidepressants going to solve the problem? Surely not..." (FG 2 female GP 45, mixed practice)*

*Quote 13*	Hiding problems	*"You hide the problem with a Band-Aid^® ^- the antidepressant - and you'll take it off after a while, saying "Oh, you're better now." (FG2, female GP, rural practice)*

ADs were seen as very useful, effective, safe and having few adverse effects, which gave the GPs a sense of self-confidence (Quote1). The GPs were not afraid to prescribe antidepressants, which were seen as having little or no risk of addiction and as not harmful (Quote 2). Though antidepressants were sometimes assessed as useless, (Quote3) they were very rarely related to suicidal intentions (Quote 4). In addition, the marketing of new categories of antidepressants (SSRIs, SNRIs) was seen as a major positive shift (Quote 5). According to the GPs, antidepressants were appropriate for a wide range of depression situations, from treating major depressive episodes to preventing the deterioration of moderate depressive conditions (Quote 6). They were not considered as useful for emergencies (Quote 7). At the beginning of the focus groups, all of the GPs, including the locums, agreed that antidepressants were rarely prescribed in situations other than those specified in the guidelines (Quote 8). The GPs expressed their opinions on the excessive number of antidepressant prescriptions by GPs, claiming that it was not malpractice (Quote 9). The participants tried to defend themselves against accusations of overprescribing antidepressants and to justify the GP posture, protecting their patients (Quote 10 & 11). In each focus group, the GPs debated the idea that their prescriptions may be seen as ineffective. They also insisted on the difficulties of prescribing antidepressants even though the patient was carefully assessed. They questioned themselves whether or not the depression was real, and whether or not prescribing an AD was truly necessary (Quote 9). When asked to explain their behaviour, the participants discussed sensitive topics such as prescribing antidepressants without any "real" diagnosis, or testing their diagnostic hypothesis with the medication. They also expressed their doubts on the reliability and usefulness of the DSM-IV criteria when assessing their patients. We will see below how GPs try to overcome these problems. Ending this part of the focus group, despite the generally positive assessments, most of the GPs insisted that these drugs had only a palliative effect (Quotes 12 & 13). Antidepressant therapy was sometimes compared to a walking stick: the prescription by itself was not enough to cure the patient.

#### Conditions (table 4)

**Table 4:** Conditions leading to prescription of an antidepressant by the GPs

Theme	Conditions n = 24
Neurology or neurological symptoms n = 8	*Isolated sleeping disorder, dementia, neuropathic pain, migraines, stroke side effects, restless legs, diffuse pain, Parkinson's disease.*
	
	*Quote 14 "I prescribe paroxetine because she has restless legs symptoms, and paresthesia ..." (FG 5, Male GP 27, locum)*

Rheumatology, Musculo- *skeletal sy*mptoms n = 5	*Fibromyalgia, lower back pain, sciatica, muscle or joint symptoms, tension headaches.*
	
	*Quote 15 "Somebody who has been coming again and again for 6 months, always complaining about lower back pain. You've tried everything, no improvement..I possibly prescribe an AD..." (FG 2 male GP, 42, rural practice)*

General symptoms n = 4	*Chronic patient, asthenia or fatigue, unexplained complaints, lack of observance among DT 2 patients.*
	
	*Quote 16 "All specialists send your patient back to you, all examinations have been done, everybody tells you there is "nothing", "nothing to be done" "(FG 4, male GP, 50, rural practice)*

Dermatology n = 1	Chronic pruritis.

Gastroenterology n = 2	*Gastrointestinal symptoms, irritable bowel syndrome.*
	
	*Quote 17 "I've had a patient, the local professor in gastroenterology prescribed him a tricyclic agent, telling it was excellent for him, and the symptoms improved a lot..." (FG 1; female GP, 49, rural practice)*

Sexual problems n = 2	Male sexual dysfunctions/impotence, premature ejaculation.
	
	*Quote 18 " I've discovered ADs can be prescribed for impotence problems" (FG 1, male GP 59, urban practice)*

Urological symptoms n = 2	Nocturnal enuresis, urinary incontinence.
	
	*Quote 19 "I prescribe ADs among patients who have post surgical incontinence, and I'm not the only one who does that. Many professors in urology do so, it's brilliant..."(FG 3; male GP, 45, urban practice)*

Although the GPs initially stated that ADs were rarely prescribed for other conditions than the guidelines [8], they mentioned 24 non-official "non-psychiatric" situations, possibly leading to prescription (Quotes 14 to 19). Symptoms (e.g. insomnia), syndromes (e.g. restless legs syndrome) or diseases (e.g. migraine) were encountered. The first group of these conditions included "standard" reasons. These are known as well recognized reasons for prescribing ADs (e.g. neuropathic pain, enuresis). The second group of conditions, less frequently described, included specific, precise conditions such as impotence or irritable bowel syndrome. Sometimes, they were the main reason for prescribing an AD. The third group included unexplained symptoms and chronic conditions with repeated complaints. An important point is that all of these medical situations were frequently combined with psychological complaints. Nevertheless, physicians felt that these symptoms were in and of themselves reasons for prescribing an AD, and not only complaints related to a major depressive episode.

### Assessing the patients and the situations

#### Coping with the patients (Table 5)

**Table 5:** Assessing the patients and the situations

Coping with the patients		
*Quote 20*	Postponing the prescription	*"Patients ask for something to get better, but I don't hear this as a request for an antidepressant, but it's difficult not to write down any prescription. Sometimes, I struggle with using "minor drugs" saying "Don't worry, it's not an antidepressant." (FG 2, female GP; 40, mixed practice)*
		
*Quote 21*		*"We do not have so many answers, feeling helpless with psychotherapy, and after that, there is only the sick note, which is not really what they are asking for"(FG 4 female GP, 41, mixed practice)*

**Coping with official criteria**		

*Quote 22*		*"When the patient has different signs, such as chronic pain, lower back pain, polymyalgia, it could be useful to deal with these issues using scales, to make the patient understand he is really in a bad situation."(Interview 3, male GP, 30, locum)*
		
*Quote23*		*"Scales are just great, scores are just great, but like you all just said: you don't use them. This kind of medicine is exactly the type we don't want to practice!"(FG 3, male GP, 59, urban practice)*
		
*Quote24*	Usefulness of scales	*When deciding what to do with a depressed patient, if I test him with a scale and there is just one point missing, I won't tell him: "Go back home, you're not depressed."(FG3, male GP, 50, urban practice)*
		
*Quote 25*		*"Some people come because they constantly feel bad, these symptoms have been with them for a long, long time. It's so complicated, there is not a real depressive episode but you can call this masked depression."(FG 4 male GP, 40, mixed practice)*
		
*Quote 26*		*"Those who work in health services, they always wait until the last minute"...or "they want a drug and a month later they say "Dr. I stopped"... "the main obstacle to treatment is the patient himself" ... "They don't want a sick note, they say they have to go to work."(FG 1, female GP, 49, rural practice)*

Patient opinions were central to initiating the prescription of antidepressants. Their initial request was usually for "something to help". Most of the GPs, including the locums, said they usually tried to postpone prescribing antidepressants by waiting and closely monitoring the patient, almost always in tandem with drugs seen as having little effect or a placebo effect (e.g. calcium, magnesium, etc.) or anti-anxiety medications (Quote 20). Some participants harshly criticized these options, accusing such drugs of masking the problem. Psychological therapy was seen as efficient but difficult to achieve due to the lack of available psychotherapists, who were seen as overworked. This therapeutic option was not suitable for everybody; some patients refused psychotherapy straightaway, soon tired of it after starting or saw it as inconvenient when coping with work hours and family commitments. On the whole, the physicians claimed they had neither the skills nor the time to perform psychotherapy by themselves. Some of them doubted the effectiveness of this kind of care, especially when social problems were prominent. As well, the GPs felt that this type of therapy was not really affordable for patients. Other options, such as giving sick notes for employees or hospitalization, were seen as short term solutions, not solving the patients' problems, and were widely rejected by the doctors (Quote 21).

#### Coping with guideline criteria (Table 5)

All of the participants insisted on the difficulty of assessing the symptoms. The GPs described a process combining guidelines and personal criteria for cases of major depression. The objective vision of the DSM-IV criteria sometimes seemed appealing. This was especially true when managing complex situations or in an educational context (Quote 22). Some GPs reported these criteria could help to support their decision. Locum physicians said that using scales on a general basis would ease the transmission of patient data to the primary-care GP they were replacing. Despite these few positive comments, the scales and recommendations were mostly criticized (Quote 23), and were considered as irrelevant in decision-making. They did not help the GPs to assess either the intensity of the depression or the usefulness of prescribing an antidepressant in real situations (Quote 24). The GPs preferred a comprehensive approach, based mainly on a patient's history, as they needed to know what was "real or not" (Quote 25). The exact proportion of "truly" depressed patients was a subject of debate. Patients were often perceived as weak, with no clear threshold between normal reactions and pathological symptoms. The way of seeking help differed from one patient to another (Quote 26). They frequently hid symptoms, and the researchers watching them during the focus group sessions analyzed their difficulties in assessing patients as being similar to playing "hide and-seek".

#### Finding solutions (Table 6)

**Table 6:** Finding solutions

*Quote 27*	Feeling	*"I feel I am able to identify him in the waiting room" (FG 2, male GP, 40, rural practice)*
Quote 28	Physician Experience	*"Our strength is our experience, which allows us to quickly diagnose, ultimately with only a few mistakes." (FG 3, male GP, 59, urban practice)*

*Quote 29*	*Knowledge of the patient*	*"I have a different threshold for my prescription if I know the patient." (FG 6, female GP, 29, locum)*

Quote 30	Contact with the patient	*"I think it depends on the relationship. A patient who is at the end of his tether and says something about it worries me less than one who keeps quiet."(FG4 female GP, 50, mixed practice)*

Quote 31	Test diagnoses	*"It's a kind of therapeutic trial: Sometimes, I get fooled. I see the patient a month later and they are doing really well. They tell me, "Hey, I stopped taking that medication." I tell myself, "If they stopped after just one month then it really wasn't a major depression."(FG1, male GP, 59, mixed practice)*

Quote 32	Calling, seeing the patient again	*"I'll call him back within 8 or 10 days to be certain of my diagnosis." "If the symptoms remain, and the patient comes back it is possible to prescribe ADs "(FG 1, male GP, 35, rural practice)*

The GPs struggled to overcome these shortcomings, searching for things such as prolonged weakness, minor symptoms (e.g. less appetite and insomnia) and multiple repeated complaints or consultations with no clearly identifiable medical reason. They also used their personal skills, basing their assessment on the patients' family, social and professional background. "Feeling", (quote 27) "experience", (quote 28) and "knowledge of the patient", (quote 29) seemed to be related. "Feeling" was described much more frequently than the other criteria, seen as an internal warning or a positive intuition. This "gut feeling" was clearly different from experience, which was related to years of medical practice. It was a quick judgment rather than a slow, meticulous compilation of "mini criteria". "Being familiar with the patient" was related to the quality of the relationship between the GP and his patient, and was more of a means to assess their personal history and the intensity of their depression symptoms (Quote 30). As a result, working with new or little-known patients proved unsettling for the GPs and locum physicians, who felt ill at ease with their own lack of effectiveness in this type of situation. More experienced GPs made a faster assessment. On the other hand, the GPs also felt that too much trust in these three components could be a pitfall leading to mistaken judgment. They sometimes used a procedure that involved giving a first prescription as a test (Quote 31) and then assessing the patient a few days later in order to check their initial feelings. This was easily done with elderly persons and with those in complex situations or having undefined symptoms (Quote 32). Doubts about the validity of this "test" were expressed.

## Discussion

This study resulted in two main key findings. The first was the GPs' prescription in numerous medical conditions not including strictly psychiatric conditions. Although we encountered AD prescription related to well-defined diseases, "combined situations" with variable combinations of physical symptoms and psychological distress were the more standard situations leading to prescription. It is already known that more than 90% of depressed patients suffer from another physical or mental disorder [19]. Although "standard causes" (e.g. depression and anxiety) were the main reasons to prescribe, the decision whether or not to prescribe was very difficult to make. Our data suggest that the GPs tried to circumvent their difficulties by developing their own tools.

The second main finding, clearly related to the first one, was a consensus on the inadequacy of the guidelines as a tool to help physicians decide whom to treat with antidepressants. The GPs developed specific skills to come up with their own personal "scales", usually based on an implicit combination of "gut feeling", "knowledge of the patient", and "small signs". This way of assessing patients has already been described [20,21]. In case of doubt, uncertainty could be diminished using the drug as a "therapeutic test". GPs also made their decision based on information about difficulties in the patients' social, professional and family lives, fatigue and repeated, unexplained requests for medical care. This way of coping with uncertainty is a strategy specific to GPs [20]. Though our GPs developed a strategy, no clear threshold for making the decision to prescribe an antidepressant was identified. This decision was clearly affected by the GPs' opinions on antidepressants, which were seen as useful drugs with no major adverse effects. The decision to prescribe was also influenced by patient's background and history, as well as by a lack of available psychotherapeutic options. In this context, a GP could view prescription of an antidepressant as "justified", even though the scale-based criteria were not met.

Our results reflected the basics of medical decision-making: combining necessity, effectiveness, safety and economy [22]. For the GPs in our sample, "necessity" meant focusing mainly on lack of recognition of depressive symptoms and under treatment, rather than useless or ineffective overtreatment. Effectiveness was seen as one of the main characteristics of antidepressants, even though the lack of practical studies in real prescribing situations has already been pointed out. Another main finding was related to the safety of antidepressants. Compared to psychological care, economy was a concern for GPs. The overall combination of these 4 factors could lead to a high antidepressant prescription rate.

### Strengths and limitations

Several points support these findings. Firstly, all of the GPs harshly criticized the guideline criteria and agreed they were irrelevant in primary care settings, thus clearly and explicitly assuming responsibility for the nature of their actual practices. From this point of view, the study's phenomenological approach was successful: the GPs recounted their true-life experiences with prescribing ADs, and the analysis was based on these experiences, not on general opinions. This was facilitated by a comprehensive approach: the participating GPs did know that this study was being conducted by GP researchers. This option was chosen in order to ensure the GPs that they would not be judged by psychiatrists: otherwise, the GPs may not have discussed their choices and difficulties so freely. Secondly, a wide variety of diagnoses, in addition to psychiatric conditions, were assessed. This leads us to believe that the GPs talked very freely about their real problems, even though this study was based on related behaviours. Finally, and despite the fact that they were younger and less experienced, the locums mostly shared the same opinions. We clearly made this choice in order to try to discover borderline therapeutic choices among GPs who would have declined the invitation to take part in a focus group on the sensitive topic of AD prescription. None of the locums recounted unexplained prescription of ADs. The choice to use a sample composed of both urban and rural GPs was also made to try to discover behaviours and difficulties related to overbooked practices or a variety of patient situations. No clear differences in their approach or behaviour appeared during the data analysis. This was the case for both younger and older, more experienced male and female GPs. This strengthens the hypothesis that our outcomes are on the whole related to primary care situations, and not only to certain practice characteristics.

Conducting focus groups on this sensitive subject could have proven to be a limitation, hiding relevant material: the GPs could have chosen to conceal odd prescriptions or decisions not covered by validated scale criteria. Nevertheless, the options chosen for the sample could not have selected doctors very interested in this topic, or on the contrary those with a strong aversion to psychiatric conditions. Another limitation is the need for more insight on the exact usefulness of the GP's personal way of assessing a patient: feelings and knowledge are well known in primary care, but we did not collect any relevant material on the way the GPs made them part of their decision-making process. One key finding was that the GPs used a personal scale, but that no data were collected to evaluate the actual performances of these scales. No evidence was gathered concerning a threshold, determined using these skills, after which an antidepressant is prescribed. Our study was not designed to explore these aspects in detail. The outcomes enable us only to make hypothesis on GPs' possibly prescribing more than necessary, and not to focus on a particular explanation. It is already well known that the positive predictive value for routine diagnosis of depression is low [18]. Could GP assessments using these personal tools increase this rate?

### Relevance for practice and future research

Another finding concerned GPs' doubts regarding the usefulness of guideline depression scales in primary care settings. In our study, we saw GPs reinventing criteria. This is consistent with other studies, showing that physicians' familiarity with a patient was an important condition in recognizing and managing depression [23]. If physicians do not see these tools as useful and do not use them at all, we should perhaps draw some conclusions: instead of making every effort to generalize the use of these tools, shouldn't we assess what is currently used in real practice? Such an assessment would likely result in practices that were more often used in everyday primary care settings. The relevance of antidepressant treatment in non-psychiatric situations in primary care seems to be a key question amongst many researchers. Various studies and trials have tested the usefulness of antidepressant drugs in "non-psychiatric" situations [24,25]. As regards primary care, the external validity of these studies is controversial, as primary care rarely involves one clear condition, but rather combines a wide variety of physical symptoms, psychological distresses and social issues. The usefulness of antidepressant treatment in various primary care settings needs to be comprehensively assessed. Prescribing fewer drugs should be considered along with making counselling and psychotherapy more available. The next step is to try and understand decision making in real settings by collecting data on antidepressant prescriptions from patient records.

## Conclusions

This study found 24 "non-psychiatric" conditions for prescribing an antidepressant in primary care and enabled a better understanding of the GPs' decision-making process. The guideline criteria officially designed to help physicians during everyday practice were found to be ineffective, thus leading the physicians to invent their own tools, to detect potential cases of depression based on their own feelings and to found their assessment of the patient on knowledge and experience.

## Competing interests

The authors declare that they have no competing interests.

## Authors' contributions

Original idea and conception of the study: AM. Development of the protocol: AM, IAA and JPL. Organization and participation in the focus groups: AM, IAA, JPL. Analysis: AM, IAA, JPL, LP. PVR and LP participated in the design and coordination and helped to draft the manuscript. Writing of the manuscript: AM. All the authors have read the draft critically, have made contributions, and have approved the final text.

## Pre-publication history

The pre-publication history for this paper can be accessed here:

http://www.biomedcentral.com/1471-2296/12/99/prepub
